# Special Issue: Recent Advances in Nanomaterials for Electrochemical Sensing and Nano-Biosensing

**DOI:** 10.3390/nano13081333

**Published:** 2023-04-11

**Authors:** Seong Jung Kwon

**Affiliations:** Department of Chemistry, Konkuk University, 120 Neungdong-ro Gwangjin-gu, Seoul 05029, Republic of Korea; sjkwon@konkuk.ac.kr; Tel.: +82-2-450-0429

Nanomaterials have been instrumental in the development of electrochemical nano-biosensors, offering high sensitivity and selectivity. By enabling miniaturization, electrochemical approaches can diagnose biomolecules in real-time and on-site. This Special Issue, entitled “Recent Advances in Nanomaterials for Electrochemical Sensing and Nano-Biosensing,” presents promising discoveries and developments in the field of electrochemical sensors that employ nanomaterials. Review articles on diagnostic methods for biosensors offer valuable insights for scientists working in this field.

Studying nanoparticles (NPs) at the single-particle level is a challenging task due to their small size and the tiny signals they generate. Rudakemwa et al. have successfully measured and interpreted the electrocatalytic properties of single palladium NPs using single-entity electrochemistry, providing a molecular-level understanding of the electrocatalytic activity and insight into its potential applications as catalysts and sensors [1]. 

Metal–organic frameworks (MOFs) are a promising class of materials for electrocatalytic applications due to their highly ordered structures, well-defined compositions, and tunable pore sizes. Xu et al. have developed an electrode coated with a MOF containing copper and tetrakis(4-carboxyphenyl) porphyrin, exhibiting excellent electrochemical and photoelectrochemical catalytic properties [2]. The MOF’s large surface area, which results from its nanostructured features, allows for the sensing of ascorbic acid in real human serum samples.

In their review article, Vaneev and colleagues discuss electrochemical nano- and microsensors that can be used to study physiological mechanisms at the cellular, tissue, and organ levels, and to investigate health and disease states [3]. The authors highlight recent advances in electrochemical sensors for measuring analytes such as neurotransmitters, oxygen, ascorbate, drugs, and pH in vivo. They also discuss the importance of antibiofouling coatings and the use of nanomaterials to improve sensor selectivity, sensitivity, and accuracy under physiological conditions.

Kim et al. provide a comprehensive overview of the current analytical methods and future perspectives of nano-biosensing technology as a platform for monitoring the state and differentiation of stem cells in stem cell engineering [4]. The authors introduce the advanced technology of surface-enhanced Raman scattering (SERS) using nanomaterials for monitoring the status of stem cells.

Molecularly imprinted polymers (MIPs) are synthetic materials that mimic natural biological antibody-antigen systems, making them suitable for use in electrochemical sensors. Dong et al. introduce nanomaterial-based MIP sensors that offer superior detection and recognition capabilities compared to conventional electrochemical sensors [5]. In particular, nanocomposites are becoming increasingly popular in the application of MIP electrochemical sensors due to their portability, sensitivity, and cost-effectiveness.

In summary, this Special Issue examines recent research trends in nanomaterial-based electrochemical nano-biosensing. Overall, these studies are working towards improving the selectivity and sensitivity of electrochemical nano-biosensors, and have the potential to drive further innovation in the field of medicine in the future.
